# Evaluation of Soluble CD48 Levels in Patients with Allergic and Nonallergic Asthma in Relation to Markers of Type 2 and Non-Type 2 Immunity: An Observational Study

**DOI:** 10.1155/2018/4236263

**Published:** 2018-09-16

**Authors:** Oded Breuer, Roopesh Singh Gangwar, Mansour Seaf, Ahlam Barhoum, Eitan Kerem, Francesca Levi-Schaffer

**Affiliations:** ^1^Department of Pediatrics, Pediatric Pulmonology Unit, Hadassah-Hebrew University Medical Center, Jerusalem, Israel; ^2^Pharmacology and Experimental Therapeutics Unit, Institute for Drug Research, School of Pharmacy, Faculty of Medicine, The Hebrew University of Jerusalem, Jerusalem, Israel

## Abstract

CD48 is a costimulatory receptor associated with human asthma. We aimed to assess the significance of the soluble form of CD48 (sCD48) in allergic and nonallergic asthma. Volunteer patients completed an asthma and allergy questionnaire, spirometry, methacholine challenge test, a common allergen skin prick test, and a complete blood count. sCD48, IgE, IL5, IL17A, IL33, and IFN*γ* were quantitated in serum by ELISA. Asthma was defined as positive methacholine challenge test or a 15% increase in FEV1 post bronchodilator in symptomatic individuals. Allergy was defined as positive skin test or IgE levels > 200 IU/l in symptomatic individuals. 137 individuals participated in the study: 82 (60%) were diagnosed with asthma of which 53 (64%) was allergic asthma. sCD48 levels were significantly elevated in patients with nonallergic asthma compared to control and to the allergic asthma cohort (median (IQR) pg/ml, 1487 (1338–1758) vs. 1308 (1070–1581), *p* < 0.01, and 1336 (1129–1591), *p* = 0.02, respectively). IL17A, IL33, and IFN*γ* levels were significantly elevated in allergic and nonallergic asthmatics when compared to control. No correlation was found between sCD48 level and other disease markers. sCD48 is elevated in nonallergic asthma. Additional studies are required for understanding the role of sCD48 in airway disease.

## 1. Introduction

CD48 is a member of the CD2 subfamily of immunoglobulin-like receptors, present as membrane receptor (mCD48) on hematopoietic cells and as a soluble form (sCD48) in serum [1–3]. CD48 functions as a costimulatory receptor which together with its ligand, CD244, is involved in numerous immune responses [2, 4]. CD48 was shown to be involved in B cell proliferation differentiation and immunoglobulin release [3], neutrophil activation [5], and T-cell stimulation and proliferation with Il-2 synthesis and receptor expression [6, 7]. Furthermore, CD48 was reported to be upregulated by viral-associated cytokines and bacterial infections [8, 9] and was shown to be involved in bacterial detection [10]. Thus, CD48 might also have a role in host defense.

Clinically, membrane and soluble CD48 expression increases under inflammatory conditions and has been shown to be elevated in patients with systemic inflammatory disorders, hematopoietic malignancies, infections, and allergic diseases [3, 8, 9, 11–13].

We have shown that CD48 participates in allergic eosinophilic airway inflammation and is a potential target for the suppression of asthma in mice, but it has also been shown that CD48 participates in nonallergic respiratory inflammatory processes [4, 8, 13–15]. We have recently found that mCD48 and sCD48 are expressed differentially in asthmatic patients of varying disease severity, sCD48 being significantly elevated in patients with mild asthma as compared to control. Moreover, mCD48 and sCD48 are both useful to differentiate mild asthma from health. Importantly patients from that study were not categorized according to their asthma phenotype, as allergic or nonallergic [16].

The multiple immune functions of CD48, its upregulation in both allergic and nonallergic, inflammatory conditions and viral and bacterial infections, have led us to speculate that sCD48 may be used as a marker for both allergic and nonallergic asthma. Previous studies assessing the significance of CD48 in asthma have not evaluated the relation to type 2 associated biomarkers and CD48 [16]. Thus, no clinical information was available about the relation of CD48 to different types of inflammatory processes in patients with asthma.

In the present study, we aimed to assess the significance of sCD48 in allergic and nonallergic asthma by comparing its expression with the expression of type 2 immunity biomarkers as well as other reported markers of allergic and nonallergic asthma.

## 2. Patients and Methods

The Institutional Committee for Human Studies approved the study (920100511), and written informed consent was obtained from all participants. Volunteer subjects from outpatient clinics in a closed homogenous community of Cochin Jews with high prevalence of asthma and allergy [17] completed an asthma and allergy questionnaire and performed spirometry with bronchodilator response, methacholine (MCH) challenge test, a common allergen skin prick test (pellitory, feathers, cat pelt, dog dander, dust mite, mold mix, *Aspergillus fumigatus*, cockroach, weed mix, southern grasses, pecan, olive, oak, eucalyptus, cypress, (Trupharm Ltd., Netanya, IL)), serum IgE levels, and a complete blood count. Patients' serum was additionally analyzed for levels of sCD48 (human CD48 ELISA kit; DEIA237; Creative Diagnostic, Shirley, NY, USA), IL17A (human IL-17A ELISA kit; 900-K84; Peprotech, Rocky Hill, NJ, USA), IL33 (human IL-33 ELISA kit; 900-K398; Peprotech, Rocky Hill, NJ, USA), IL5 (human IL-5 ELISA kit; 430405; BioLegend, San Diego, CA, USA), and IFN*γ* (human IFN*γ* ELISA kit; 88731688; Affymetrix, eBioscience, San Diego, CA, USA). Asthma severity was not assessed in the current study due to its cross-sectional design.

Asthma was defined as positive MCT or 15% increase in FEV1 post bronchodilator administration in symptomatic individuals [18]. Allergy was defined as positive skin prick test for at least one common allergen or IgE levels > 200 IU/l in symptomatic individuals [19], and allergic asthma was defined as having both asthma and allergy. Data was summarized by grouping the subjects as healthy controls (no evidence of asthma or allergy or any other disease) or having allergic or nonallergic asthma; patients suffering from allergy without evidence of asthma were excluded from cytokine analysis.

Results were presented as medians and interquartile range (IQR) for continuous variables and percentages for nominal variables. For bivariate comparisons, of two continuous variables, *T*-test in case of normal distribution and the Mann-Whitney test for nonnormal distributions were used. For multiple comparisons of continuous variables, the ANOVA test was used with the Tukey's correction and the Kruskal-Wallis test in case of nonnormal distributions. For nominal variables, the Chi-square test or Fisher exact test was used where necessary. Correlations between two quantitative variable parameters were tested by computing Spearman's rank coefficients (*r*).

The discriminative ability of the sCD48 levels to predict a diagnosis of asthma was assessed using ROC curve analysis. All statistical analyses were performed using GraphPad Prism 6 (La Jolla, CA, USA). All ELISA experiments were performed in triplicate.

## 3. Results

One hundred and ninety-seven individuals participated in the study with a median age of 40 years (23.0–50.0) (range 5–79); 113 (57%) were female, and 82 (41%) were diagnosed with asthma of which 53 (64%) were allergic asthma; 60 volunteers had evidence of allergy without asthma and were excluded from cytokine analysis; 55 (40%) volunteers who did not meet criteria for allergy or asthma and were otherwise healthy were included as a control group. Patient characteristics are presented in Table 1.

**Figure 1:**
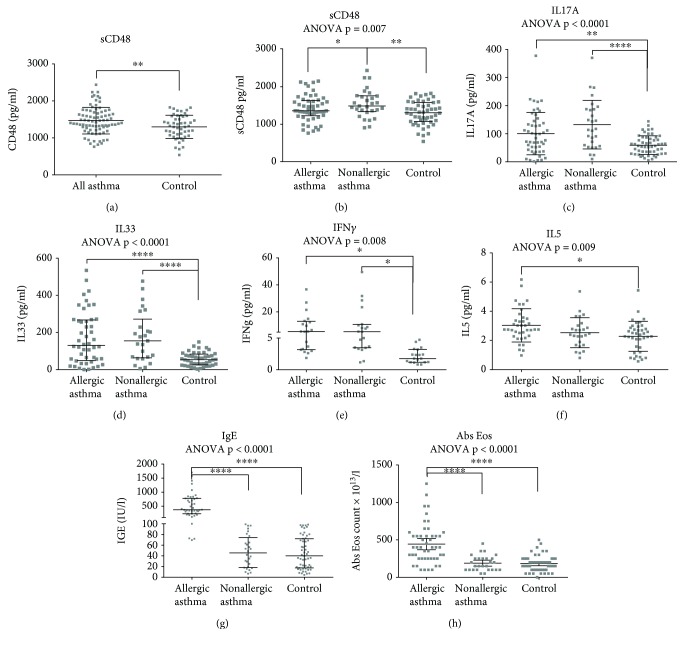
sCD48 in volunteers with asthma and control (a), sCD48 (b), cytokines (c–f), IgE (g), and absolute eosinophil numbers (h) in volunteers with allergic asthma, nonallergic asthma, and control. Data are shown as the median (IQR); ^∗^*p* < 0.05, ^∗∗^*p* < 0.01, ^∗∗∗^*p* < 0.001, and ^∗∗∗∗^*p* < 0.0001. Abs: absolute; Eos: eosinophil.

Levels of sCD48 were significantly elevated in patients with asthma when compared to control (median (IQR) pg/ml, 1416 (1262–1693) vs. 1308 (1070–1581), respectively, *p* < 0.01) (Figure 1).

After subgrouping asthma patients according to their disease phenotype (allergic or nonallergic asthma), sCD48 levels were still significantly elevated only in patients with nonallergic asthma compared to control and to the allergic asthma cohort (median (IQR) pg/ml, 1487 (1338–1758) vs. 1308 (1070–1581), *p* < 0.01, and 1336 (1129–1591), *p* = 0.007, respectively) (see Figure 1 and Table 2).

**Figure 2:**
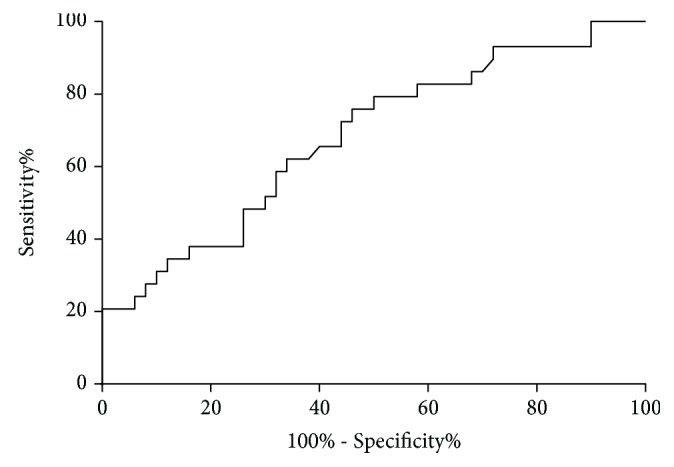
Receiver operator characteristic curves (ROCs) of sCD48 to predict nonallergic asthma. Area under the ROC curve = 0.68 (95% CI, 0.56–0.80, *p* = 0.008).

The area under the ROC of sCD48 for identifying nonallergic asthma was 0.68 (95% confidence interval (CI) 0.56–0.80, *p* = 0.008) indicating a low-moderate predictive power (Figure 2).

As shown in Figure 1, IL17A, IL33, and IFN*γ* levels were significantly elevated in allergic and nonallergic asthmatics when compared to control, whereas IL5 levels were elevated only in patients with allergic asthma when compared to control.

sCD48 levels were not correlated with any type 2 biomarkers or any other cytokine evaluated (Table 3). Furthermore, sCD48 levels were not related to patients' sex (*p* = 0.6), smoking history (yes/no) (*p* = 0.65), asthma medication (as grouped by stepwise GINA approach (*p* = 0.6) [18], or any type of chronic medical treatment (*p* = 0.33).

## 4. Discussion

We have conducted a cross-sectional observational study in volunteer patients from a community with high prevalence of asthma and allergy [17]. The main finding in this study is that sCD48 levels are mildly but significantly elevated in patients with nonallergic asthma and that sCD48 has a low to moderate predictive value for the diagnosis of nonallergic asthma.

The roles of CD48 as a costimulatory receptor of various immune responses, its involvement in allergen-induced airway inflammation, and its regulation in mice by ORMDL3 have strongly implicated CD48 association to human asthma [4, 14, 20]. Indeed, previous studies have shown that CD48 controls activation of both human and mouse eosinophils and that in a mouse model of allergic airway inflammation, anti-CD48 antibodies dramatically reduce airway inflammation [8]. Our hypothesis in the current study was that sCD48 levels will reflect atopic sensitization and will be elevated in patients with allergic asthma and possibly also reflect neutrophilic airway inflammation and thus be elevated also in patients with nonallergic asthma.

The results of this study not only show that sCD48 was not significantly elevated in patients with allergic asthma but also that sCD48 levels were not correlated to any marker of type 2 immunity. An explanation for this may lay in the difference between systemic and localized allergic inflammatory responses. Similarly, we have previously found diminished levels of sCD48 and of mCD48 on peripheral blood leukocytes in patients with atopic dermatitis [21] and allergic rhinitis (unpublished results, Eliashar et al.). However, an upregulated CD48 response was found in tissues of these patients. Thus, it is possible that CD48 is not systemically upregulated in allergic inflammation but only in tissues where the inflammatory reaction takes place [13, 21]. Unfortunately, the protocol of this study did not include biomarkers in sputum and thus we cannot comment on airway sCD48 upregulation in allergic asthma. A different explanation for this could lie in the differences between mouse and human lung physiology. The evidence supporting the involvement of CD48 in allergic diseases comes mainly from murine models for asthma. However, differences between mice and men exist in innate and adaptive immunity as well as in lung structure and function. Mice do not spontaneously develop asthma, and sensitization is used to achieve T cell differentiation toward Th2 phenotype. Moreover, cytokines in mice which control skewing of T cell differentiation toward Th1 or Th2 phenotypes are not entirely similar to human cytokines. Furthermore, although the existence of polarized T cell populations with different cytokine responses is an observed disease paradigm in mice, in humans, this distinction between a Th1 and Th2 response is not always so clear. Thus, mice models cannot fully mimic the complexity of human asthma phenotypes and especially of what is regarded as nonallergic asthma [22, 23]. The involvement of sCD48 in nonallergic asthma may be related to its more general role in inflammatory processes. CD48 has numerous roles in the regulation of immunity and has been shown to be elevated in patients with hematopoietic malignancy, infections, and inflammatory diseases with autoimmunity [1, 2, 4, 6, 12]. Recently, CD48 was shown to be associated with pulmonary inflammation in patients with systemic sclerosis [15]. Furthermore, previous studies have shown that neutralization of CD48 significantly reduced proinflammatory cytokine expression [8] thus elevated sCD48 levels in patients with nonallergic asthma may presumably be related to an increased inflammatory state. In the current study, the inflammatory cytokines, IL17A, IL33, and IFN*γ*, were elevated in serum of patients with both allergic and nonallergic asthma, reflecting an active inflammatory state in all our asthmatic patients. We have evaluated these cytokines due to the fact that in recent years these cytokines have been demonstrated to be involved in the regulation of airway inflammation and asthma. Increased serum IL17A and IL33 levels were found in patients with various degrees of asthma as well as steroid-resistant and neutrophilic asthma [24–32]. We regarded these cytokines as potential markers that will help clarify CD48's role in airway inflammation in asthma and as markers that may help assess the degree of inflammation in our patients. Unfortunately, despite the fact that IL17A, IL33, and IFN*γ* were all elevated in patients when compared to control, sCD48 levels had no relation to any of these cytokine levels. Thus, even though CD48 seems to be involved in airway inflammation in patients with nonallergic asthma, its exact inflammatory function is still unclarified. We hypothesize that CD48 participates in cell activation in response to infection and inflammation via interactions between CD48 and CD244 on mast cells, eosinophils, T-cells, and basophils. sCD48 may be released from these cells post activation and thus might have a role in the regulation of the cellular response.

We have previously shown that sCD48 levels are increased mainly in patients with mild asthma when compared to control [16]. Therefore, we expected to find reduced sCD48 levels in patients with more severe asthma. Interestingly, we did not find any significant correlation between sCD48 levels and other patient characteristics, such as sex, smoking, chronic medication, and medication for asthma. Patients receiving high doses of inhaled corticosteroids or oral corticosteroids did not have significant lower levels of sCD48. Furthermore, cigarette smoking which has been previously shown to have harmful effects on asthma possibly due to underlying inflammatory mechanisms [33] was also not associated with sCD48 levels. Nevertheless, this study was not powered to assess different correlations in these subgroups of patients; therefore, we cannot definitely conclude on these results.

Our study has several disadvantages. First, the cross-sectional nature of the study precludes the ability to evaluate sCD48 levels in different stages of disease and response to treatment and its longitudinal significance. Second, as mentioned above, the lack of sputum prevents us from commenting on localized sCD48 response in patients with allergic and nonallergic asthma. Third, this study also lacks the analysis of membrane-bound CD48 which might have assisted in the understanding of the cellular pathways involved.

The main strength of our study lies in the careful classification of patients as suffering from asthma or allergy. This objective distinction between asthma phenotypes strengthens the validity of our results clinically.

## 5. Conclusions

In conclusion, in this study, we have found that sCD48 levels are elevated in the serum of patients with nonallergic asthma. The significance of the elevated sCD48 levels is not fully understood. It may be related to airway inflammation and cellular activation. We suggest to asses sCD48 levels in patients with other inflammatory respiratory conditions.

## Figures and Tables

**Table 1:** Patient characteristics.

	All	Allergic asthma	Nonallergic asthma	Control	*p* value
Subjects, *n*	137	53	29	55	—
Gender (M/F)	47/90	22/31	12/17	13/42	0.1
Age, years, mean (SD)	37.93 (19.38)	37.42 (22.25)	34.17 (22.2)	40.42 (14.12)	0.47
Smoking (yes/no)	26/137	8/45	3/26	15/40	0.11
Current use of inhaled or oral corticosteroid^#^*n* (%)	23 (16.7)	16 (30.2)	7 (25.1)	0 (0)^∗^	<0.001

^#^As reported by patients at the time of assessment. ^∗^Significantly lower.

**Table 2:** sCD48, type 2 biomarkers, and cytokine levels according to patient categorization (see also Figure 1).

	Allergic asthma (*n* = 53)	Nonallergic asthma (*n* = 29)	Control (*n* = 55)	ANOVA *p* value
sCD48 (pg/ml)	1358 (1228–1632)^∗^	1487 (1338–1758)^∗^/^∗∗^	1308 (1070–1581)^∗∗^	0.007
IgE (IU)	374.5 (233.8–781.5)^∗∗∗∗^	45.5 (18.25–74.15) ^∗∗∗∗^	40 (17.5–72.1) ^∗∗∗∗^	<0.0001
Eos count	400 (250–550)^∗∗∗∗^	150 (100–250)^∗∗∗∗^	175 (100–250)^∗∗∗∗^	<0.0001
IL5 (pg/ml)	2.746 (2.31–3.51)^∗^	2.434 (1.68–3.06)	2.395 (1.52–2.86)^∗^	0.009
IL17A (pg/ml)	84.44 (39.97–141.6)^∗∗^	115.5 (56.8–187.3)^∗∗∗∗^	56.7 (29.28–84.07)^∗∗^/^∗∗∗∗^	<0.0001
IL33 (pg/ml)	130.3 (49.46–267.5)^∗∗∗∗^	154.9 (63.58–271.9)^∗∗∗∗^	54 (25.73–83.78)^∗∗∗∗^/^∗∗∗∗^	< 0.0001
IFNg (pg/ml)	5.475 (3.19–13.05)^∗^	5.338 (3.47–10.68)^∗^	1.768 (1.18–3.2)^∗^/^∗^	0.008

Data is presented as median (IQR); ^∗^*p* < 0.05, ^∗∗^*p* < 0.01, and ^∗∗∗∗^*p* < 0.0001.

**Table 3:** Correlations between sCD48 levels and type 2 biomarkers and evaluated inflammatory cytokines.

	sCD48 all volunteers	sCD48 in allergic asthma	sCD48 in nonallergic asthma	sCD48 in control
IL33 (pg/ml)	0.02 (−0.17–0.21), *p* = 0.8	−0.17 (−0.44–0.13), *p* = 0.2	0.09 (−0.33–0.48), *p* = 0.7	0.06 (−0.25–0.36), *p* = 0.7
IL17 (pg/ml)	0.09 (−0.09–0.27), *p* = 0.3	−0.19 (−0.46–0.09), *p* = 0.2	0.049 (−0.34–0.42), *p* = 0.8	0.09 (−0.19–0.36), *p* = 0.5
IFN*γ* (pg/ml)	0.21(−0.06–0.45), *p* = 0.1	−0.08 (−0.52–0.39), *p* = 0.7	0.41 (−0.05–0.73), *p* = 0.07	−0.19 (−0.60–0.30), *p* = 0.4
IgE (pg/ml)	0.06 (−0.13–0.25), *p* = 0.5	0.27 (−0.04–0.54), *p* = 0.08	−0.23 (−0.56–0.17), *p* = 0.2	−0.13 (−0.4–0.14), *p* = 0.3
Eos Abs (pg/ml)	0.11 (−0.07–0.28), *p* = 0.2	0.25 (−0.04–0.50), *p* = 0.06	−0.14 (−0.50–0.26), *p* = 0.5	0.03 (−0.24–0.31), *p* = 0.8
IL5 (pg/ml)	−0.04 (−0.24–0.16), *p* = 0.7	−0.08 (“-0.4–0.26), *p* = 0.6	0.30 (−0.10–0.62), *p* = 0.1	−0.3 (−0.57–0.03), *p* = 0.06

Data presented as Spearman correlation coefficients (IQR), *p* value.

## Data Availability

The database with results of this study is available by request from the Pharmacology and Experimental Therapeutics Unit, Institute for Drug Research, School of Pharmacy, Faculty of Medicine, The Hebrew University of Jerusalem, Jerusalem, Israel, and Department of Pediatrics, Pediatric Pulmonology Unit, Hadassah-Hebrew University Medical Center, Jerusalem, Israel.

## Abbreviations

sCD48: Soluble CD48
mCD48: Membrane-bound CD48
MCT: Methacholine challenge test.
